# A meta-analysis of the success rates of heartbeat restoration within the platinum 10 min among outpatients suffering from sudden cardiac arrest in China

**DOI:** 10.1186/s40779-016-0071-8

**Published:** 2016-03-22

**Authors:** Xiang-Min Gu, Zhi-Hui Li, Zhong-jie He, Zhe-Wei Zhao, Shuang-Qing Liu

**Affiliations:** School of Graduate, Liaoning Medical College, Jinzhou, Liaoning 121001 China; Department of ICU, First Affiliated Hospital, General Hospital of PLA, Beijing, 100036 China

**Keywords:** Cardiac arrest, Cardiopulmonary resuscitation, Meta-analysis, Platinum 10 minutes, Time-efficiency, First aid

## Abstract

**Background:**

The optimal time to save a person who has had a sudden cardiac arrest is within the first few minutes of the incident. Early compression and early defibrillation should be performed at this time. Timeliness is the key to successful CPR; as such, Prof. He proposed the “platinum 10 min” system to study early CPR issues. This paper systematically evaluates the success rates of heartbeat restoration within the “platinum 10 min” among patients suffering from sudden cardiac arrest.

**Methods:**

The clinical data of outpatients suffering from a cardiac arrest were retrieved from the China Knowledge Network (January 1975–January 2015), the Chongqing VIP database (January 1989–January 2015), and the Wanfang database (January 1990–January 2015). The success of the cardiopulmonary resuscitation (CPR) performed at different times after the patients had cardiac arrests was analyzed. Two researchers screened the literature and extracted the data independently. A meta-analysis was conducted using Stata12.0. A total of 57 papers met the inclusion criteria, including 29,269 patients. Of these patients, 1776 had their heartbeats successfully restored. The results showed high heterogeneity (*X*^*2*^ = 3428.85, *P* < 0.01, I^2^ = 98.4 %). The meta-analysis was conducted using a random-effects model. The combined effect size was 0.171 (0.144–0.199).

**Results:**

(1) The success rate of heartbeat restoration did not differ among the four emergency treatment methods that patients received: the methods described in the 2000 Guidelines for CPR and Emergency Cardiovascular Care, that described in the 2005 version, 2010 version, and another CPR method. (2) The patients were divided into five groups based on the time when CPR was performed: the ≤1 min group, the 1- ≤ 5 min group, the 5- ≤ 10 min group, the 10- ≤ 15 min group and the >15 min group. The CPR success rates of these five groups were 0.247 (0.15–0.344), 0.353 (0.250–0.456), 0.136 (0.109–0.163), 0.058 (0.041–0.075), and 0.011 (0.004–0.019), respectively. The CPR success rates did not differ between the patients in the ≤1 min group and the 1- ≤ 5 min group. This success rate was higher for the patients in the 1- ≤ 5 min group than those in the 10- ≤ 15 min group, those in the 10- ≤ 15 min group, and those in the >15 min group. The CPR success rate was higher for the patients in the 5-10 min group than those in the 10- ≤ 15 min group and those in the >15 min group.

**Conclusions:**

The CPR success rate was higher for the patients in the 10- ≤ 15 min group than those in the >15 min group. In addition, the patients were divided into two groups based on whether CPR was performed within the first 10 min after the cardiac arrest occurred: the ≤10 min group and the >10 min group. The CPR success rate was higher for the patients in the ≤10 min group (0.189 [0.161–0.218]) than those in the >10 min group (0.044 [0.032–0.056]). (3) Differences were not found between the CPR success rates among the patients in the telephone guidance group (0.167 [0.016–0.351]) and those in the ≤1 min, 1- ≤ 5 min, 5- ≤ 10 min, 10- ≤ 15 min, and >15 min groups. (4) The CPR success rates did not differ among in the patients in the witness + public group (0.329 [0.221–0.436]), those in the ≤1 min group, and those in the 1- ≤ 5 min group. However, this success rate was higher in the patients in the witness + public group than those in the 5- ≤ 10 min, 10- ≤ 15 min, and >15 min groups.

**Conclusions:**

The success rate of heartbeat restoration did not differ among patients receiving CPR based on different guidelines. The success rate of CPR lies in its timeliness. The participation of the general population is the cornerstone of improving CPR. Providing complete emergency treatment equipment and perfecting comprehensive measures can improve the success rate of CPR among patients within the platinum 10 min. CPR research in China must be improved.

## Background

Cardiopulmonary resuscitation (CPR) is the most important medical method of saving patients who experience cardiac and respiratory arrest. More than 70 % of cardiac arrests occur outside the hospital [1]. The life of a patient who has recently experienced cardiac and respiratory arrest can be saved when correct CPR methods are employed. The annual number of deaths due to sudden cardiac arrest in China is greater than 2,500,000. These deaths cause immense damage to the patients’ families and society. The optimal time to save a person who has had a sudden cardiac arrest is within the first few minutes of the incident. Early compression and early defibrillation should be performed at this time [2]. Timeliness is the key to successful CPR; as such, Prof. He proposed the “platinum 10 min” system to study early CPR issues. This term refers to the importance of performing CPR within 10 min after a patient has had a cardiac arrest [3]. The difficulty in collating the clinical CPR data in China limits the objective evaluation of the current situation. The present study conducted a meta-analysis of the CRP papers published in the Chinese literature from 1979 to 2015 and investigated the success rates of early CPR to provide relevant data to improve this technique in China.

## Data and methods

### Inclusion and exclusion criteria

To be included in the current study, the papers must have addressed studies of CPR in which the patients had their heartbeats restored using the methods discussed in different international versions of the Guidelines for CPR and Emergency Cardiovascular Care. The included papers were retrospective case summaries and analyses. Only papers written in Chinese were included. The included papers investigated outpatients who had cardiac arrests caused by various diseases and who received CPR within a specific time frame. These papers included a specific number of patients who recovered (i.e., had a spontaneous heart rhythm and a restored pulse) after CPR. Papers were excluded from this study when the number of studied patients was less than or equal to 20. Duplicate and unpublished reports were also excluded.

### Retrieval strategy

The China Knowledge Network (January 1979–January 2015), the Wanfang database (January 1990–January 2015), and the Chongqing VIP database (January 1989–January 2015) were used to search for and retrieve papers with Chinese keywords such as “心跳骤停 + 院前(cardiac arrest + out of hospital)” and “心肺复苏 + 院前(CPR + out of hospital)” in their titles or section titles.

### Paper screening, data extraction, and quality evaluation

Papers were screened using the above inclusion and exclusion criteria. Two researchers independently read the titles and abstracts of the obtained papers. After eliminating papers that did not meet the inclusion criteria, these researchers read the full text of the papers to determine whether they met the inclusion criteria. If the two researchers disagreed with regard to whether a paper met the inclusion criteria, then they discussed their decisions to come to consensus or asked a third party to determine whether the paper should be included in the study. The content of the included papers contained the following: (1) general information (title, authors, publication date, and paper source); (2) research characteristics (whether specific times existed when the CPR was performed, whether a specific number of patients were saved, and whether a specific number of deaths was recorded); and (3) at least one type of the following clinical outcome data: data regarding whether the patients had recovered a pulse, spontaneous respiration, or consciousness and data regarding patient discharge.

### Statistical analyses

Analyses were conducted using Stata 12.0. The chi-square test was used to test data heterogeneity. P-values of >0.05 denoted no significant heterogeneity in the dependent variables. A meta-analysis was conducted using a fixed-effects model. P-values of <0.05 denoted heterogeneity in the dependent variables. A random-effects model can be used when heterogeneity remains after controlling for it but when the combined data continue to have clinical significance [4]. The I^2^ statistic was used to reflect the magnitude of the heterogeneity of the combined effect. Larger I^2^ values denote greater heterogeneity. If I^2^ < 25 %, then mild heterogeneity exists across the studies. If 25 % < I^2^ < 75 %, then moderate heterogeneity exists across the studies. If I^2^ > 75 %, then high heterogeneity exists across the studies [5]. A funnel plot was used to estimate the publication bias. A two-sided test was used when a need existed to test the above statistical analyses. The significance level was set at 0.05.

## Results

### Literature retrieval

**Fig. 1:**
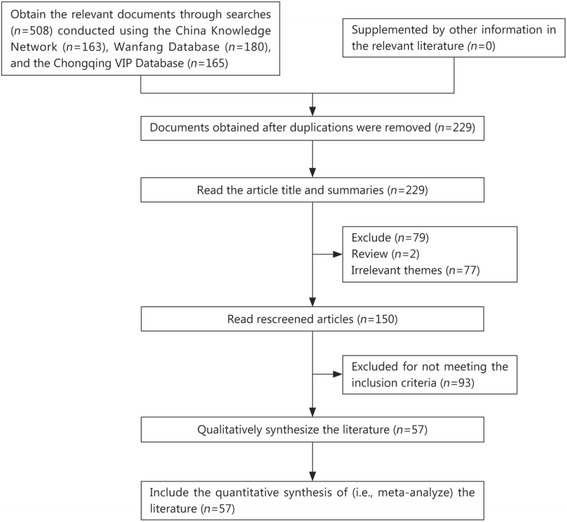
Flowchart of literature screening

A total of 508 relevant papers were initially retrieved. After screening the papers, 57 papers were included in the final analysis [6–62]. A total of 29,269 patients were included in these 57 papers. Figure 1 shows a flow chart of the literature screening.

### General information of the papers

**Fig. 2:**
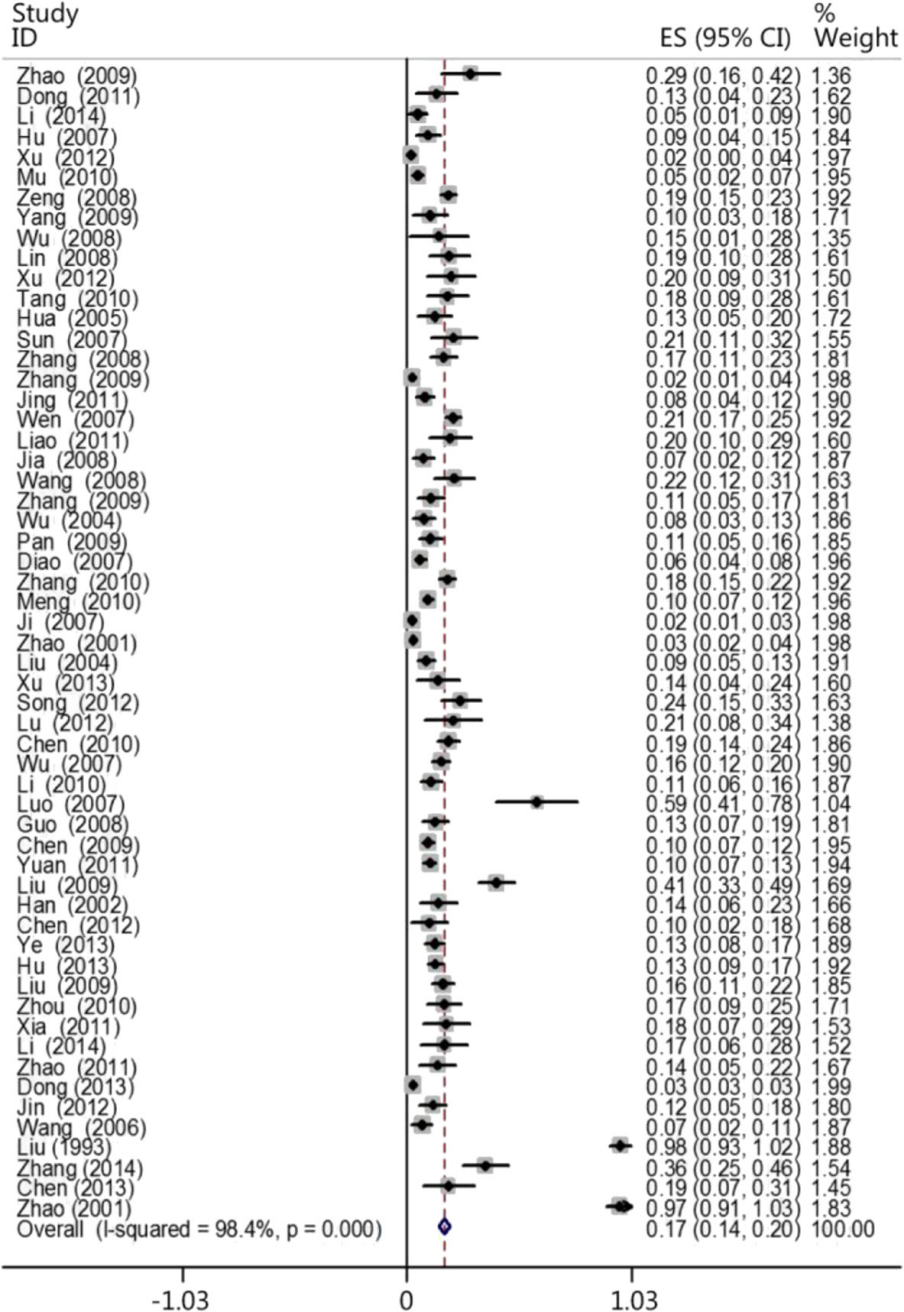
CPR success rates

**Fig. 3:**
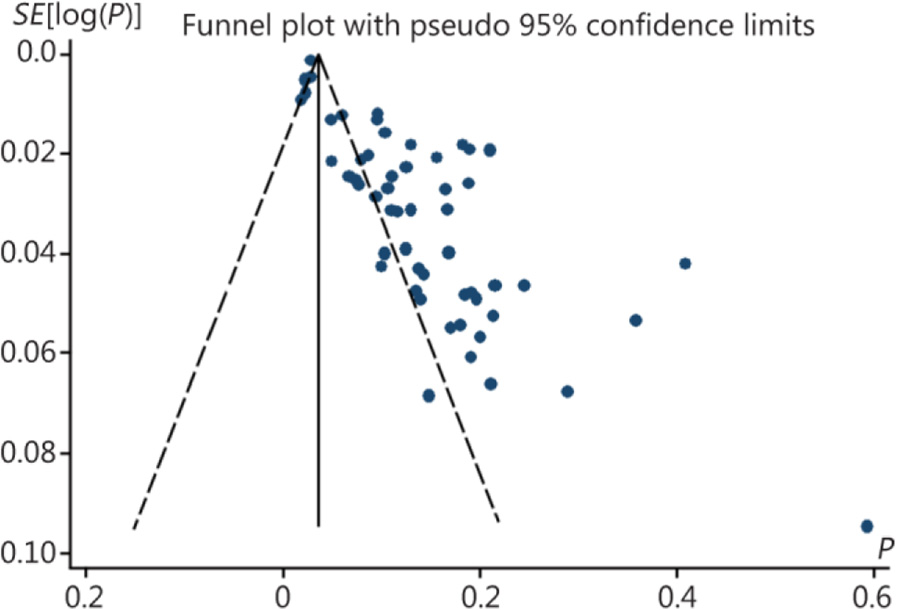
Funnel plot of the publication bias

Exactly 57 papers met the inclusion criteria, containing 29,269 patients. Of these patients, the heartbeats of 1776 patients were restored. The results of the heterogeneity test (*X*^2^ = 3428.85, *P* < 0.01, I^2^ = 98.4 %) indicated high heterogeneity. A meta-analysis was conducted using a random-effects model. The combined effect size was 0.171 (0.144–0.199; Fig. 2). The papers selected for analysis showed a publication bias (Fig. 3).

### CPR was performed using the methods provided by different guidelines

**Fig. 4:**
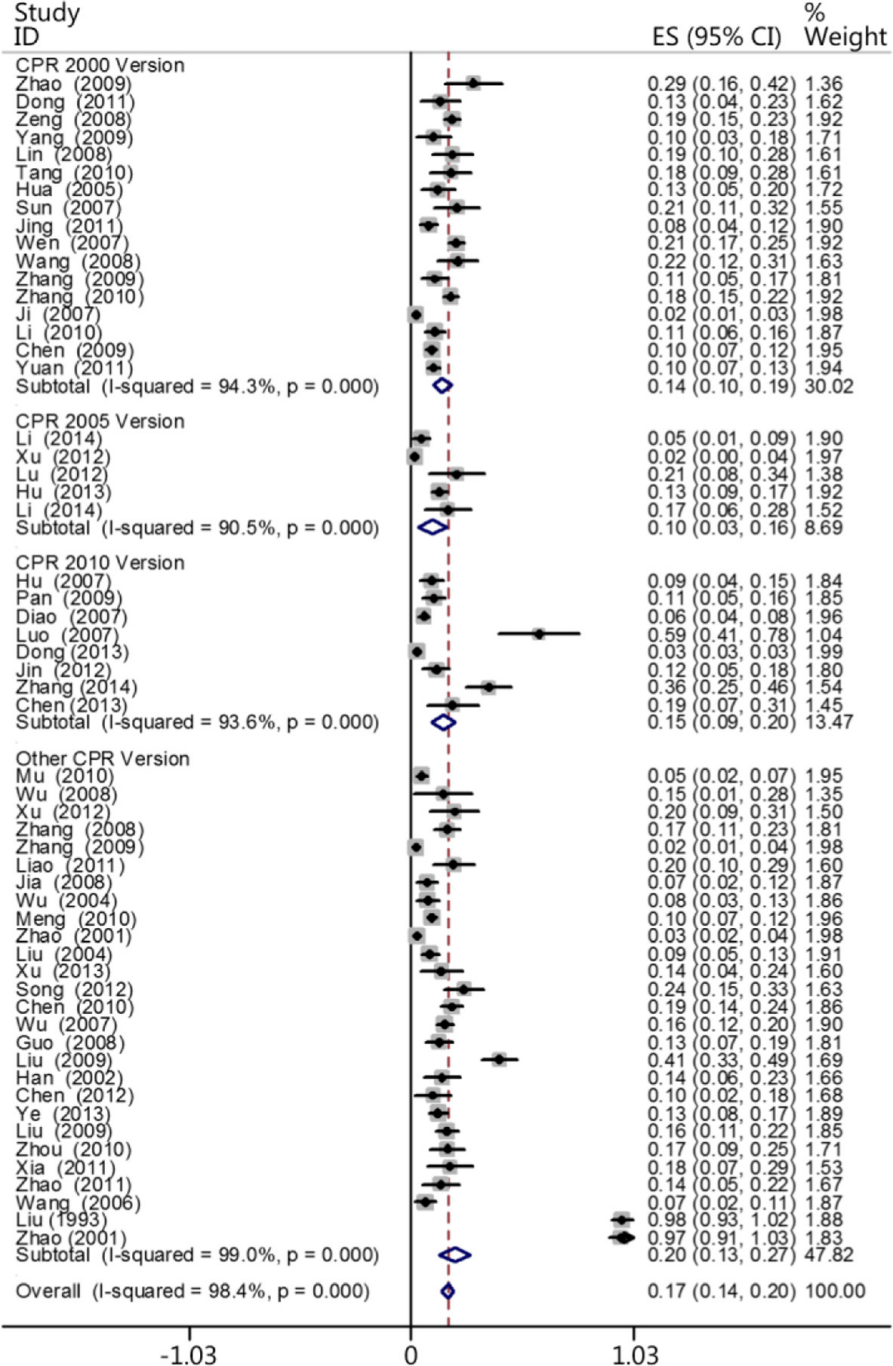
Comparisons of the CPR success rates using the methods provided by the different guidelines

The CPR methods applied in the reviewed 57 papers include the methods listed in the 2000 Guidelines for CPR and Emergency Cardiovascular Care (CPR 2000) as well as those in the 2005 and 2010 versions and another version. Totals of 17, 5, 8, and 27 papers used the methods in the 2000, 2005, 2010, and other guidelines, respectively. The results showed that the heartbeat restoration success rate using the 2000 version (0.145 [0.103–0.186]), 2005 version (0.099 [0.034–0.164]), 2010 version (0.147 [0.092–0.201]), and other version (0.202 [0.130–0.274]) did not significantly differ (Fig. 4). This result does not eliminate the possibility that timeliness is the most important factor affecting CPR success rate (Table 1).

**Table 1:** Comparisons of the CPR success rates using the methods provided across the different guidelines

Guidelines	Case	Heterogeneity test			Model	Success rate	Confidence Intervals	Number of patients	Number of successes
		Q-value	P-value	I^2^ (%)					
2000 Version	17	279.33	<0.01	94.3	Random	0.145	0.103–0.186	3936	495
2005 Version	5	42.20	<0.01	90.5	Random	0.099	0.034–0.164	751	70
2010 Version	8	109.10	<0.01	93.6	Random	0.147	0.092–0.201	19,513	636
Other Version	27	2671.57	<0.01	99.0	Random	0.202	0. 130–0.274	5069	575
Total	57	3428.85	<0.01	98.4	Random	0.171	0.144–0.199	29,269	1776

CPR 2000 Version: 2000 Guidelines for CPR and Emergency Cardiovascular Care; CPR 2005 Version: 2005 Guidelines for CPR and Emergency Cardiovascular Care; CPR 2010 Version: 2010 Guidelines for CPR and Emergency Cardiovascular Care; Other Version: The methods are not be included in 2000, 2005 and 2010 version

### Timeliness of CPR

**Fig. 5:**
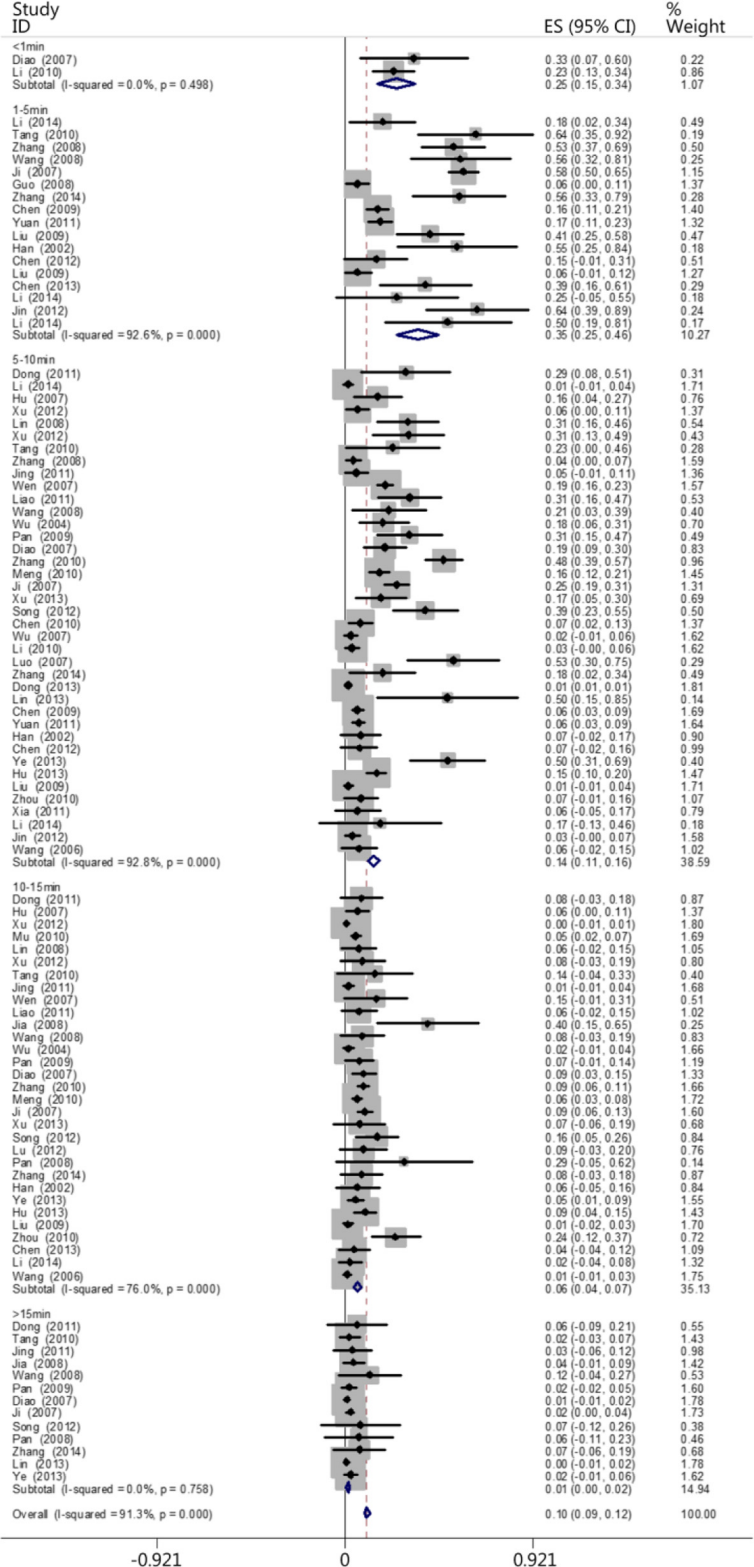
Comparison of the CPR success rates at different times

**Fig. 6:**
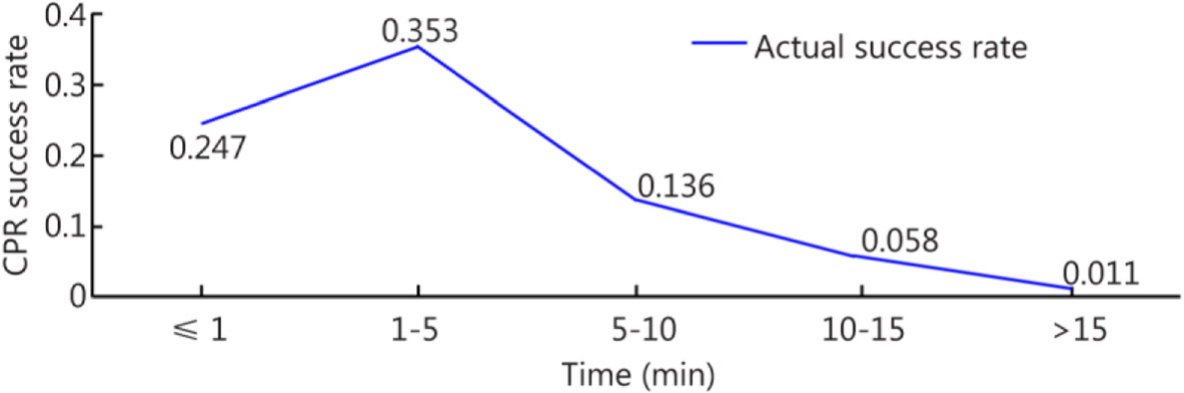
Actual heartbeat restoration time curve within the platinum 10 min

**Fig. 7:**
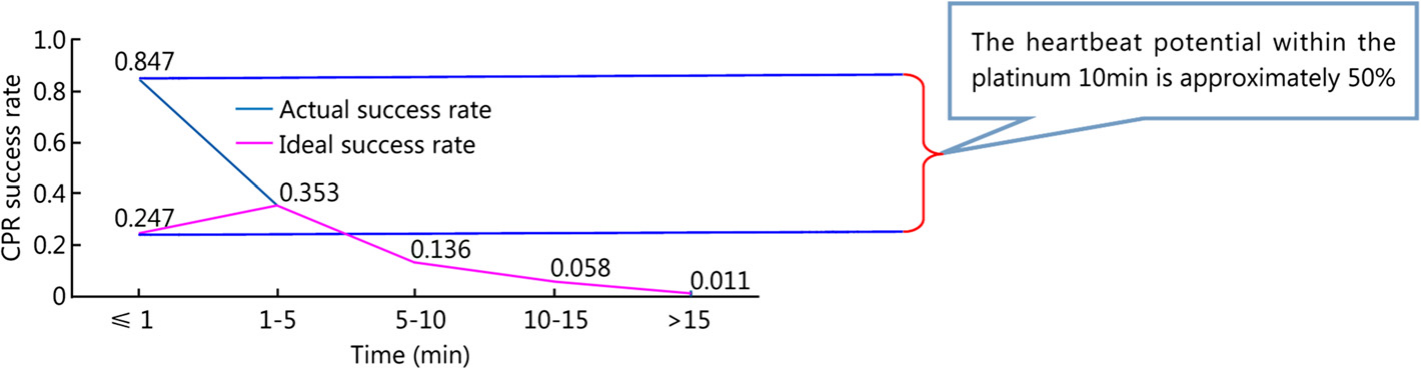
Ideal heartbeat restoration time curve within the platinum 10 min

The patients were divided into five groups based on the time when each patient received CPR: the ≤1 min group, the 1- ≤ 5 min group, the 5- ≤ 10 min group, the 10- ≤ 15 min group, and the >15 min group. Two, 17, 39, 31, and 13 of the reviewed papers included patients who belonged to the ≤1 min, 1- ≤ 5 min, 5- ≤ 10 min, 10- ≤ 15 min, and >15 min groups, respectively. The results showed that the survival rate decreased as the interval between cardiac arrest and CPR increased. The heartbeat restoration success rate for the ≤1 min group (0.247 [0.15–0.344]) did not differ from that of the 1- ≤ 5 min group (0.353 [0.250–0.456]). This success rate was higher for patients in the 1- ≤ 5 min group (0.353 [0.250–0.456]) than those in the 5- ≤ 10 min (0.136 [0.109–0.163]), 10- ≤ 15 min (0.058 [0.041–0.075]), and >15 min groups (0.011 [0.004–0.019]). This success rate was higher for the patients in the 5- ≤ 10 min group (0.136 [0.109–0.163]) than those in the 10- ≤ 15 min (0.058 [0.041–0.075]) and >15 min groups (0.011 [0.004–0.019]). This success rate was higher for the patients in the 10- ≤ 15 min group (0.058 [0.041–0.075]) than those in the >15 min group (0.011 [0.004–0.019]; Fig. 5). Table 2 shows the results. Figures 6 and 7 show the actual and ideal success rates of the heartbeat restoration time curves within the platinum 10 min. From these curves, we infer that the difference between the ideal and actual values is 0.600 (0.847–0.247; i.e., a 60 % treatment potential exists).

**Table 2:** Comparison of the CPR success rates at different times

Group	Case	The test for heterogeneity			Model	Success rate	Confidence Intervals	Number of patients	Number of successes
		Q-value	P-value	I^2^ (%)					
≤1 min	2	0.46	0.498	0	Random	0.247	0.151–0.344	76	19
1- ≤ 5 min	17	216.92	0.00	92.6	Random	0.353	0.250–0.456	839	254
5- ≤ 10 min	39	529.82	0.00	92.8	Random	0.136	0.109–0.163	19,825	654
10- ≤ 15 min	31	124.98	0.00	76.0	Random	0.058	0.041–0.075	2516	171
>15 min	13	8.34	0.758	0	Random	0.011	0.004–0.019	719	13
Total	102	1160.59	0.00	91.3	Random	0.105	0.092–0.118	23,975	1111

**Fig. 8:**
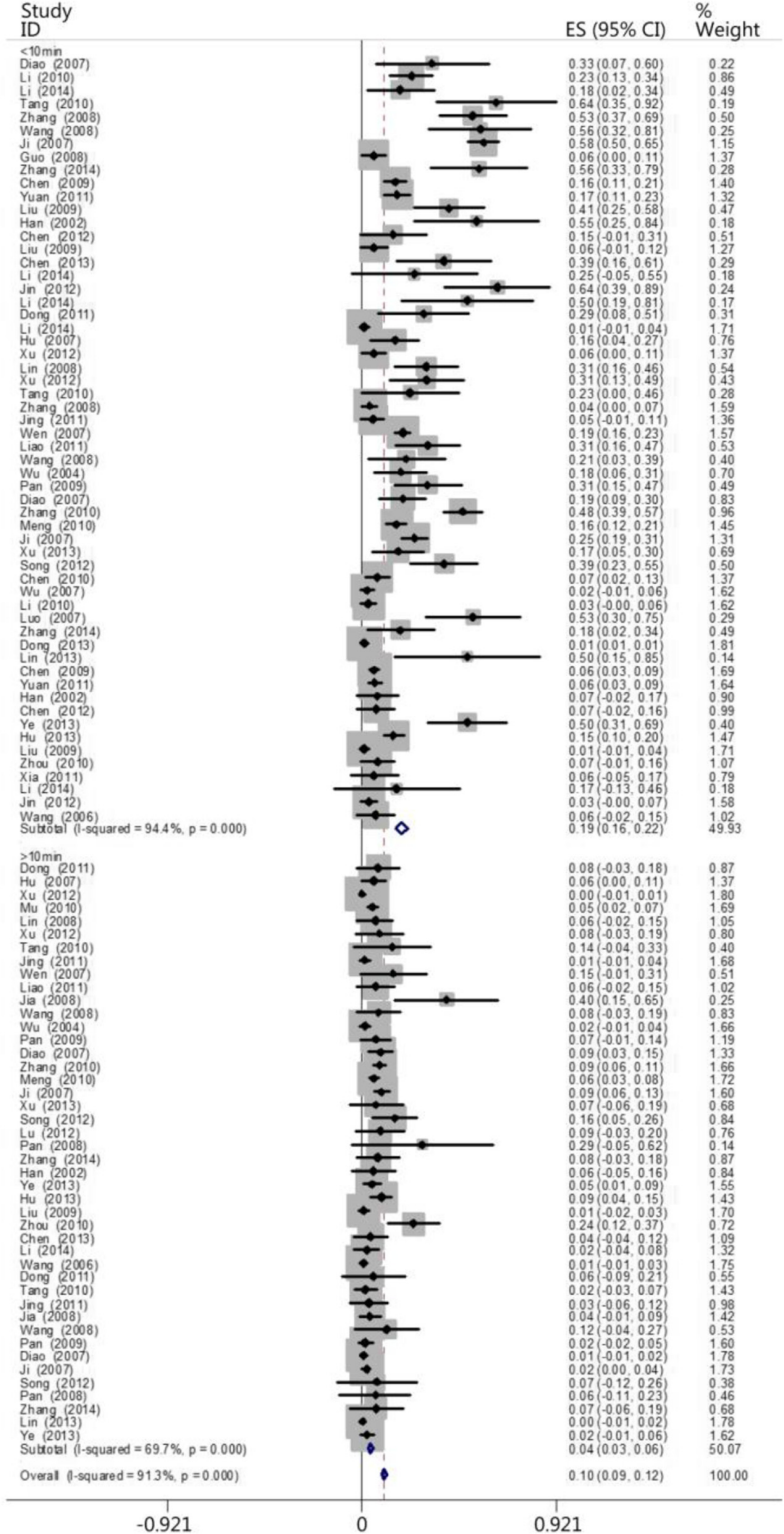
The CPR success rates for the ≤10 min and >10 min groups

**Fig. 9:**
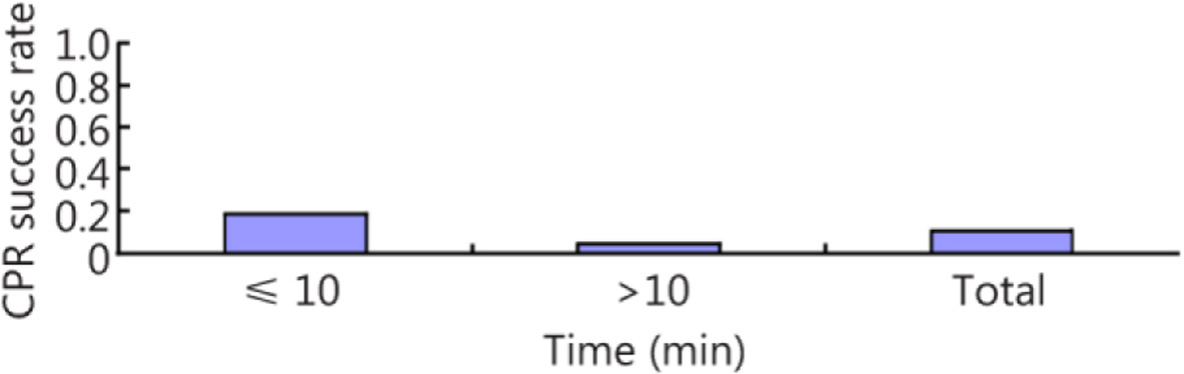
A histogram of the CPR success rates for the ≤10 min and >10 min groups

The patients were divided into two groups based on the time when CPR was performed: the ≤10 min group and the >10 min group. According to the times recorded in the papers, 58 papers included patients who belonged to the ≤10 min group, and 44 papers included patients who belonged to the >10 min group. The results showed that the heartbeat restoration success rate was higher for the patients in the ≤10 min group (0.189 [0.161–0.218]) than those in the >10 min group (0.044 [0.032–0.056]; Fig. 8; Table 3). The ratio of successful heartbeat restorations in the ≤10 min group to that in the >10 min group was 4.3: 1 (Fig. 9).

**Table 3:** The CPR success rates in the ≤10 min and >10 min groups

Group	Case	Heterogeneity test			Model	Success rate	Confidence intervals	Number of patients	Number of successes
		Q-value	P-value	I^2^ (%)					
≤10 min	58	1014.96	<0.01	94.40	Random	0.189	0.161–0.218	20,740	927
>10 min	44	141.71	<0.01	69.7	Random	0.044	0.032–0.056	3235	184
Total	102	1160.59	<0.01	91.3	Random	0.105	0.092–0.118	23,975	1111

**Fig. 10:**
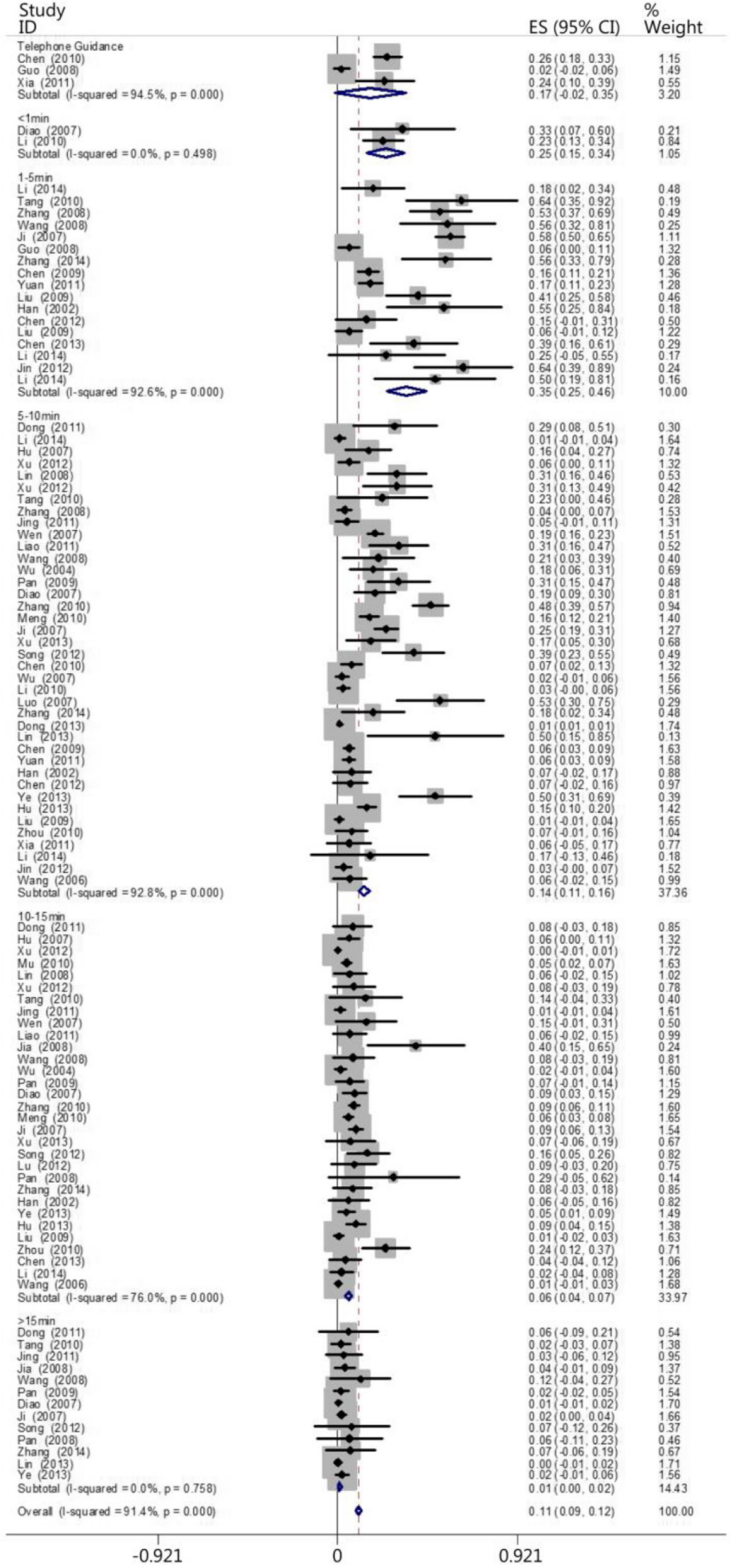
The CPR success rates of the telephone guidance group and the other groups at different times

**Fig. 11:**
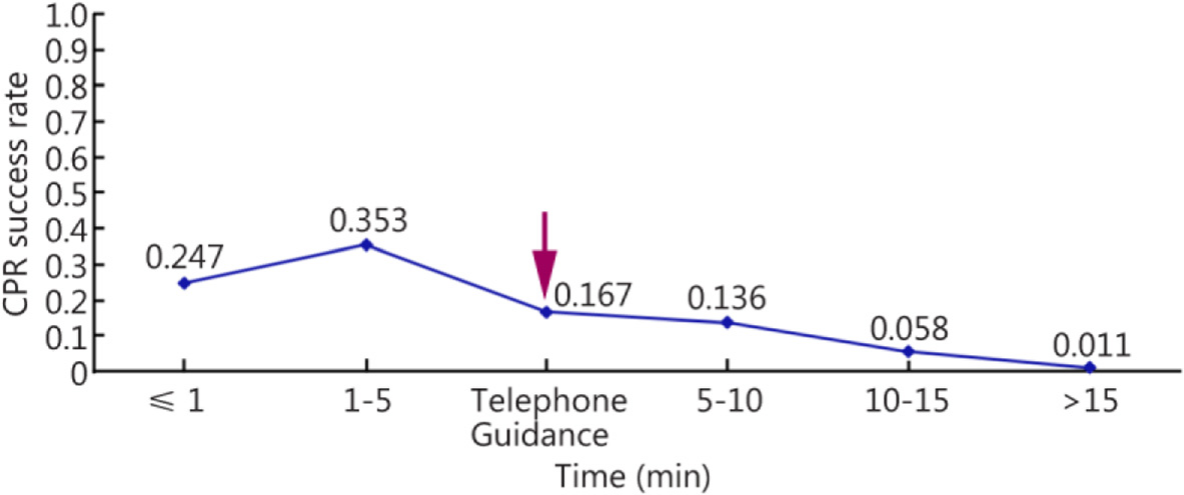
Line chart of the CPR success rates of the telephone guidance group and the other groups at different times

The patients were also divided into groups based on whether the emergency treatment was provided under the guidance of a medical professional over a telephone (the telephone guidance group) and then compared with the ≤1 min, 1- ≤ 5 min, 5- ≤ 10 min, 10- ≤ 15 min, and >15 min groups. Three, two, 17, 39, 31, and 13 papers included patients who belonged to the telephone guidance, ≤1 min, 1- ≤ 5 min, 5- ≤ 10 min, 10- ≤ 15 min, and >15 min groups, respectively. The results showed that the heartbeat restoration success rates for the telephone guidance group (0.167 [0.016–0.351]), the ≤1 min group (0.247 [0.151–0.344]), the1- ≤ 5 min group (0.353 [0.250–0.456]), the 5- ≤ 10 min group (0.136 [0.109–0.163]), the 10- ≤ 15 min group (0.058 [0.041–0.075]), and the >15 min group (0.107 [0.004–0.019]) did not differ (Fig. 10, Table 4). We believe that the CPR success rate associated with the telephone guidance group should be equivalent to that of the 1- ≤ 5 min group. However, more papers must be included to make this determination. Figure 11 compares the heartbeat restoration success rates of all groups. The arrow indicates the location of the heartbeat restoration success rate associated with the telephone guidance group on the timeliness curve.

**Table 4:** The CPR success rates of the telephone guidance group and the other groups at different times

Group	Case	Heterogeneity test			Model	Success rate	Confidence Intervals	Number of patients	Number of successes
		Q-value	P-value	I^2^ (%)					
Telephone guidance	3	36.22	0.00	94.5	Random	0.167	0.016–0.351	226	46
≤1 min	2	0.46	0.498	0	Random	0.247	0.151–0.344	76	19
1- ≤ 5 min	17	216.92	0.00	92.6	Random	0.353	0.250–0.456	839	254
5- ≤ 10 min	39	529.82	0.00	92.8	Random	0.136	0.109–0.163	19,825	654
10- ≤ 15 min	31	124.98	0.00	76.0	Random	0.058	0.041–0.075	2516	171
>15 min	13	8.34	0.758	0	Random	0.107	0.004–0.019	719	13

**Fig. 12:**
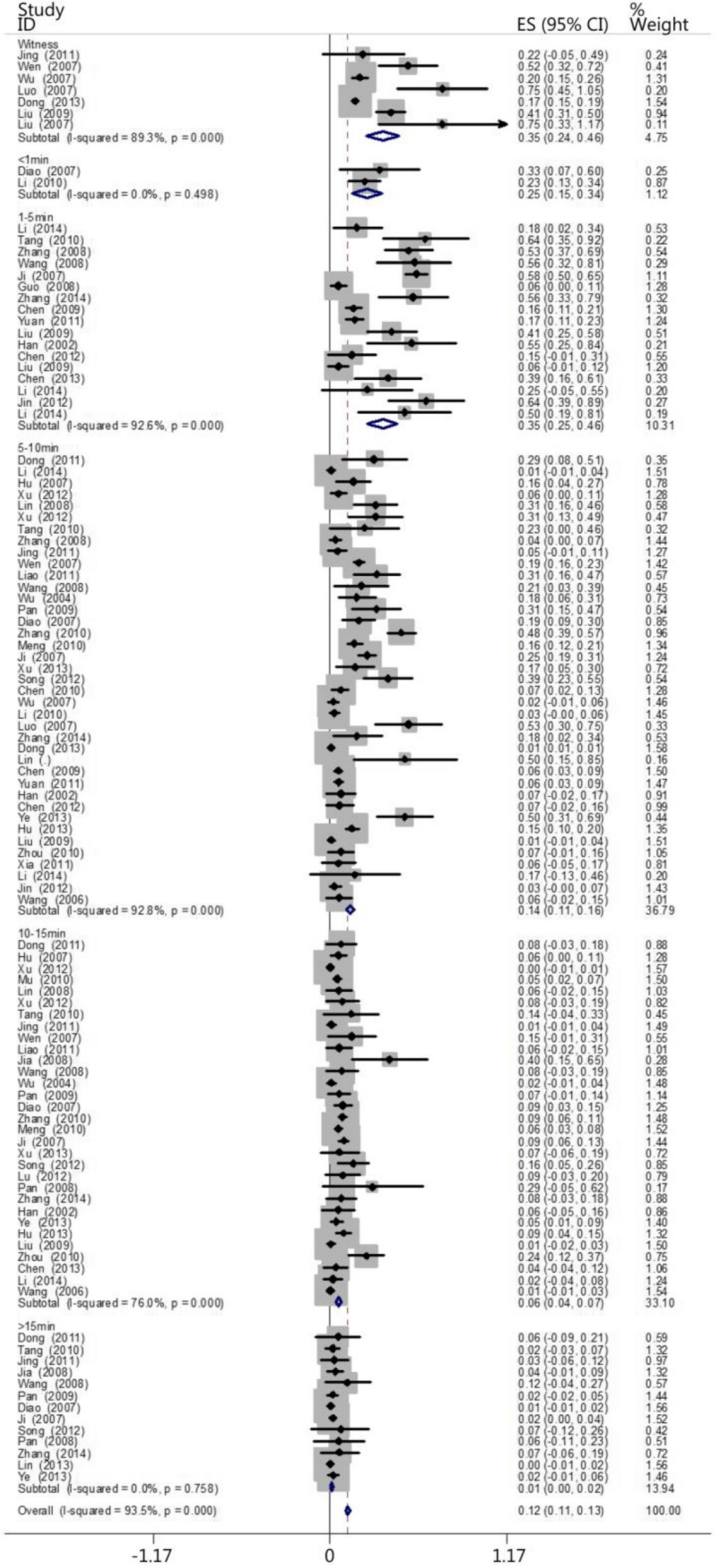
The CPR success rates of the witness + public group and the other groups at different times

**Fig. 13:**
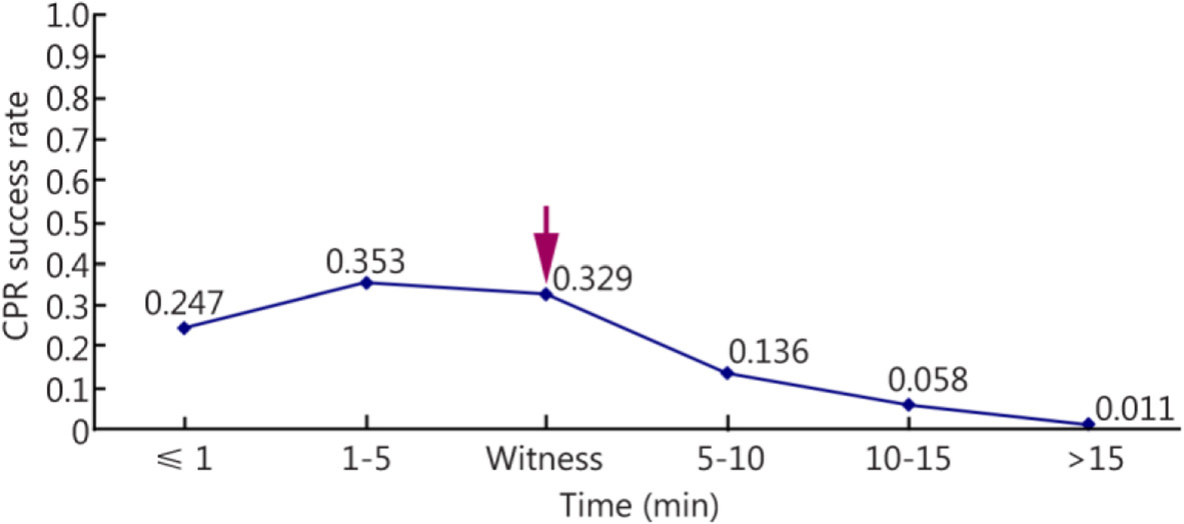
Line chart of the CPR success rates of the witness + public group and the other groups at different times

The patients were also divided into the following groups based on whether a witness was present and if people in the public saved the patient (the witness-public group referred to the person who found the patients and rescued them in the first time) and then compared with the ≤1 min, 1- ≤ 5 min, 5- ≤ 10 min, 10- ≤ 15 min, and >15 min groups. According to the times recorded, seven, two, 17, 39, 31, and 13 papers included patients who belonged to the witness + public, ≤1 min, 1- ≤ 5 min, 5- ≤ 10 min, 10- ≤ 15 min, and >15 min groups, respectively. The results did not reveal differences among the heartbeat restoration success rates of the witness + public group (0.329 [0.221–0.436]), the ≤1 min group (0.247 [0.151–0.344]), and the 1- ≤ 5 min group (0.353 [0.250–0.456]). However, this success rate was higher for the witness + public group (0.329 [0.221–0.436]) than the 5- ≤ 10 min group (0.136 [0.109–0.163]), the10- ≤ 15 min group (0.058 [0.041–0.075]), and the >15 min group (0.011 [0.004–0.019]; Fig. 12; Table 5). Figure 13 shows the location of the witness + public group on the timeliness curve. This result suggests that the strategy of advocating that people witness cardiac arrests to save one’s self and others is a step in the right direction.

**Table 5:** The CPR success rates of the witness + public group and the other groups at different times

Group	Case	The test for heterogeneity			Model	Success rate	Confidence Intervals	Number of patients	Number of successes
		Q-value	P-value	I^2^ (%)					
Witness	7	49.32	0.00	89.9	Random	0.329	0.221–0.436	2245	432
≤1 min	2	0.46	0.498	0	Random	0.247	0.151–0.344	76	19
1- ≤ 5 min	17	216.92	0.00	92.6	Random	0.353	0.250–0.456	839	254
5- ≤ 10 min	39	529.82	0.00	92.8	Random	0.136	0.109–0.163	19,825	654
10- ≤ 15 min	31	124.98	0.00	76.0	Random	0.058	0.041–0.075	2516	171
>15 min	13	8.34	0.758	0	Random	0.011	0.004–0.019	719	13

## Discussion

Out-of-hospital cardiac arrests remain a major event that affects public health worldwide. Their rate of occurrence has increased from 37 per 100,000 people to 121 per 100,000 people [63]. Studies have shown that many factors affect CPR outcomes, including the time needed to recover spontaneous circulation, the patient’s initial heart rate, their age, additional complications, and so forth [64, 65]. A 2011 survey conducted in the US showed that the survival rate of patients who had suffered from a cardiac arrest and received out of hospital CPR was 26.3 %, and the eventual discharge rate was 9.6 % [66]. The limitation of the papers included in the present study affects the analysis of CPR in other aspects. Therefore, we investigated the outcomes of CPR within the “platinum 10 min” to emphasize the results of Chinese research. We extracted a single clinical index from the data (i.e., heartbeat, which is recovered first during the CPR process) to analyze the effectiveness of early CPR. This analysis might partially reflect the status of CPR in China. In particular, we comparatively analyzed factors such as the early participation of the public and its effect, which has practical and historical significance for CPR in China.

Past researchers have been divided concerning whether time, technique, or personnel is the most important aspect regarding CPR. The present study compared four emergency treatment methods: the 2000 CPR version, the 2005 CPR version, the 2010 CPR version, and another CPR version. No differences were observed with regard to CPR success rate across these guidelines. Therefore, once a person has mastered the basic CPR technique (this person is not required to acquire the same level of CPR training as professional medical staff), timeliness is the most important factor that affects its success rate [67, 68].

Another concept exists with regard to CPR: “the gold 4 min”. Most theorists believe that patients who receive CPR within 4 min after cardiac arrest have a survival rate of 50 %; those who receive CPR between 4 and 6 min, between 6 and 10 min, and after 10 min following cardiac arrest have survival rates of only 10 %, 4 %, and almost zero, respectively [1]. Shortcomings exist when 4 min is considered as the gold standard for CPR success and was set as the target because patients who receive CPR within 4 min of cardiac arrest have a survival rate of only 50 % according to a theoretical analysis. If we desire success rates such as 60, 70, 80, or 90 %, then when should CPR be performed? Without the participation of the general public, this timeliness will not occur.

The “platinum 10 min” timeliness pattern for CPR stresses that every minute and second before the arrival of medical staff is important. We strive to reach a success rate of 100 %. The current study showed that the CPR success rate of the patients in the ≤1 min group did not differ from that of the 1–5 min group. However, this success rate was higher among patients in the 1–5 min group than those in the 5–10 min, 10–15 min, and >15 min groups. This success rate was higher among the patients in the 5–10 min group than those in the 10–15 min and >15 min groups. This success rate was higher among the patients in the 10–15 min group than those in the >15 min group. This success rate was significantly higher among the patients in the ≤10 min group than those in the >10 min group. More papers and patients must be included to further investigate timeliness patterns. Based on the results of the present study, however, we can conclude that the success rate of CPR lies in its timeliness. The success rate of heartbeat restoration increases as the gap between cardiac arrest and CPR decreases.

We propose a “platinum 10 min” strategy; specifically, it is necessary to perform chest compressions on a patient who suffers from a cardiac arrest each minute over the first 10 min after cardiac arrest. The comparative analysis of the CPR success rates for the patients in the telephone guidance, ≤1 min, 1–5 min, 5–10 min, 10–15 min, and >15 min groups did not find a difference among the patients from the first three groups. This result might be because relatively few papers and patients were included in the present study, and the difference in the CPR success rates among these three groups was not shown. In addition, no differences were found between the CPR success rates of the patients in the witness + public group and those in the ≤1 min and 1–5 min groups. However, this success rate was higher among the patients in the witness + public group than those in the 5–10 min, 10–15 min, and >15 min groups. This success rate was higher among the patients in the witness + public group than those in the telephone guidance group, which indicates that the prospect of the general public performing CPR for those who have had a cardiac arrest matches expectations. This conclusion is now the theoretical basis for placing the duty/responsibility of performing CPR for cardiac arrest on the public.

The present study has certain limitations. (1) All of the 57 papers included in the present study are retrospective, observational studies. Large differences exist in the quality of their clinical data. It is impossible to comprehensively evaluate CPR with regard to heartbeat, respiration, brain function, and living condition. (2) The funnel plot indicates that these papers have a publication bias; this conclusion affects the accuracy of this meta-analysis and suggests that actual CPR success rates are lower than those reported in the present study.

## Conclusions

Timeliness is the core of CPR. The strategy based on the time-efficiency is “self- and mutual-saving”. “Self- and mutual-saving” refers to the behavior shown when the wounded/sick or the public saves the wounded/sick at the site of the incident before the arrival of medical professionals. Self- and mutual-saving is the start and foundation of all treatments, and it is as important as or even more important than professional treatment. Self- and mutual-saving increases optimal treatment timeliness and the maximal timeliness value; moreover, it is the initial link on the emergency-treatment chain. The quality of self- and mutual-saving directly determines the overall treatment effect [69]. We should encourage people to learn emergency treatment techniques. The success rate of CPR will improve as more people master this technique.

## Abbreviations

CPR: Cardiopulmonary resuscitation

## References

[1] Wang YT, Li DX, Lin GF (1992). Practical emergency medicine.

[2] Huang ZT (2004). Improve the level of cardiopulmonary resuscitation measures and countermeasures. Chin J Emerg Med.

[3] He ZJ (2012). More on aid platinum ten minutes. Med J Chin PLA.

[4] Zintzaras E, Ioannidis JPA (2005). Hegesma: Genome search meta-analysis and heterogeneity testing. Bioinformatics.

[5] Ioannidis JP, Patsopoulos NA, Rothstein HR (2008). Research methodology: Reasons or excuses for avoiding meta-analysis in forest plots. BMJ.

[6] Zhao HL (2002). 13 cases of cardiopulmonary resuscitation rescue and nursing. Hebei Med.

[7] Pan HB (2008). 15 cases of sudden cardiac resuscitation before analysis stop hospital. World Health Digest.

[8] Lu YB, Wei YF (2012). Experience in 38cases of pre-hospital resuscitation of patients with cardiac arrest caused by electrical injury. J Youjiang Med Univ Natl.

[9] Chen WZ (2012). Clinical analysis of 50 cases of cardiopulmonary resuscitation before hospital. Chin Pract Med.

[10] Dong BY, Yang LL (2011). 52 cases of sudden death in patients with pre-hospital emergency cardiopulmonary resuscitation analysis. Chin J Modern Drug Appl.

[11] Li XL, Zhang SH (2014). 102 patients before hospital cardiopulmonary resuscitation retrospective analysis. Sichuan Med J.

[12] Hu JC, Liu XM, Wang XD (2007). 106 cases of cardiac and respiratory arrest patients before hospital CPR analysis. General Pract Med.

[13] Liu CH, Zhao ZG, Wu GF (2009). Clinical analysis of 188 cases of the former hospital cardiopulmonary resuscitation. Proc Clin Med.

[14] Xu J (2012). 218 cases of cardiopulmonary arrest cardiopulmonary resuscitation before stopping hospital clinical analysis. Sichuan Med J.

[15] Mu LH, Xu B, Sun H (2010). Clinical analysis of 266 cases of the former hospital cardiopulmonary resuscitation and discuss. Healthc Forum.

[16] Zeng YH, Yin JZ, Pan M, Liang QL (2008). Clinical analysis of 428 cases of the former hospital cardiopulmonary resuscitation. Intern Med Health Guid.

[17] Chen GH (2010). Telephone guidance CPR application of pre-hospital care. Shandong Med J.

[18] Yang HF, Jia JH (2009). Recovery 58 cases of respiratory and cardiac arrest prior to analysis hospital cardiopulmonary. Gansu Med J.

[19] Wu HC (2002). 27 cases of emergency cardiopulmonary resuscitation. Chin J Crit Care Med.

[20] Li XQ (2014). 47cases of pre-hospital factors CPR success rate analysis. Clin Res.

[21] Xu YY (2013). On the hospital before the ambulance in different situations CPR for cardiac arrest affect the patient's. Road Health Mag.

[22] Li SQ (2010). Witnesses on the scene to rescue the impact of pre-hospital cardiopulmonary resuscitation success rate. Chin Remedy Clin.

[23] Liu P, Liu GL, Zu LJ (2004). Pediatric hospital emergency first aid and cardiopulmonary resuscitation clinical analysis. Chin J Coal Ind Med.

[24] Seerhazi Y (2013). Site of the former hospital CPR rescue analysis of patients with sudden death. Med Inform.

[25] Lin LF (2008). The influence factor pre-hospital emergency cardiopulmonary resuscition success rate. Today Nurs.

[26] Liu X (2007). Tianjin prehospital CPR success rates causes. Chin J Urban Rural Enter Hyg.

[27] Xia LJ (2011). "120" telephone instructions in CPR application of pre-hospital care[J]. Chin J Pract Nurs.

[28] Xu ZB, Chen YX (2012). Analysis of factors affecting pre-hospital emergency cardiopulmonary resuscitation. Jilin Med J.

[29] Yuan QM, Li FM, Shi XQ (2011). CPR related factors influence the success or failure of the hospital prior to analysis. Chin J Gen Med.

[30] Tang FR, Yang T, Huang W (2010). Analysis of 65 cases of pre-hospital emergency cardiopulmonary resuscitation. Chin Mod Med.

[31] Hua HM (2010). 72 cases of pre-hospital cardiopulmonary resuscitation and clinical analysis. Chin J Emerg Res Dis Med.

[32] Xi J, Deng YJ, Qu Z (2012). A retrospective analysis of 103 cases of pre-hospital cardiopulmonary resuscitation to success. World Health Digest.

[33] Sun LP (2007). 61 cases of pre-hospital cardiac arrest rescue breathing experience. Proceed Clin Med.

[34] Zhao LX, Li JH, Wang F (2011). Sudden death in patients with pre-hospital emergency cardiopulmonary resuscitation (CPR) 65 cases. Front Med.

[35] Zhang JY, He SJ (2008). First aid CPR 144 cases. Mod Med Health.

[36] Zhang Y (2009). Success of 9 cases of pre-hospital emergency cardiopulmonary resuscitation. Shanxi Med J.

[37] Jing XM (2011). The importance of pre-hospital emergency cardiopulmonary resuscitation success rate of exploration and medical fit. Chine Med Innov.

[38] Zhou BL (2010). Pre-hospital emergency cardiopulmonary resuscitation success rate Causes. Pract J Cardiac Celeb Pneum Vascu Dis.

[39] Wang XD (2006). Pre-hospital emergency cardiopulmonary resuscitation in 105 patients. Fujian Med J.

[40] Wen CM (2007). Prehospital CPR, retrospective analysis of 452 cases. Med J Liaoning.

[41] Hu JH, Liu XM, Yang C, Wang Z, Zhang WT, Zhang Q (2013). Pre-hospital emergency cardiopulmonary resuscitation different conditions affect the efficacy of cardiac arrest patients. J Anhui Health Vocat Tech College.

[42] Liao WQ (2011). Factors pre-hospital emergency cardiopulmonary resuscitation analysis. Chin Foreign Med Res.

[43] Jia YR, Wang PY (2008). Pre-hospital emergency medical care with CPR. J Pract Med Tech.

[44] Luo QX (2007). Pre-hospital cardiopulmonary resuscitation 27 cases. J Pract Med.

[45] Wang GF (2008). Pre-hospital cardiopulmonary resuscitation analysis of 79 cases. Chin J Misdiag.

[46] Zhang Y, Xiao H (2009). Clinical analysis of 100 cases of pre-hospital cardiopulmonary resuscitation[J]. Mod Med J Chin.

[47] Wu X, Lin SB (2004). 115 cases of pre-hospital cardiopulmonary resuscitation analysis. Lingnan J Emerg Med.

[48] Pai YY (2009). Clinical analysis of 132 cases of pre-hospital cardiopulmonary resuscitation. Shandong Med J.

[49] Diao PS (2007). 382 cases of pre-hospital cardiopulmonary resuscitation correlation analysis. Mod J Integ Tradit Chin West Med.

[50] Zhang M, Hua HM, Gong X (2010). 454 cases of pre-hospital cardiopulmonary resuscitation success factors and intervention strategies. Med Chin Fam Physicians.

[51] Chen X (2009). 504 cases of pre-hospital cardiopulmonary resuscitation cardiac and respiratory arrest patients. Chin Med Guide.

[52] Meng H (2010). Pre-hospital cardiopulmonary resuscitation retrospective analysis of 605 cases. J Chin Tradit Chin Med Inf.

[53] Ji YJ (2007). Pre-hospital cardiopulmonary resuscitation retrospective analysis of 796 cases. Chin J Crit Care Med.

[54] Zhao YT (2001). Pre-hospital cardiopulmonary resuscitation analysis of 36 cases. Henan J Diagn Ther.

[55] Wu YL, Zhang XY, Wang CM (2007). Discussion success and failure CPR prehospital. Proc Clin Med.

[56] Liu WL, Li SZ, Liu SF (2004). Explore the success factors of pre-hospital cardiopulmonary resuscitation. Am J Chin Clin Med.

[57] Guo QX, Li Y (2008). Explore ways of pre-hospital CPR. Chin Com Doctor.

[58] Han XT (2002). Correlation factors pre-hospital cardiopulmonary resuscitation. Shandong Med J.

[59] Song QQ, Zhou XH, Cheng J, Li CM, Wang HB (2012). Specification CPR for sudden death in patients with pre-hospital emergency rescue observed in. Pract J Card Cereb Pneumal Vasc Dis.

[60] Liu WM (1993). Suzhou City, the pre-hospital cardiopulmonary resuscitation analysis of 41 cases. J Emerg Med.

[61] Dong J, Lu F (2013). Influence "2010AHA Guide" of the former Shanghai hospital cardiopulmonary resuscitation success rate. Healthy People.

[62] Zhao JH, Zhang JY (2001). Pre-hospital cardiopulmonary resuscitation analysis of 34 cases. Hebei Med.

[63] Boyd TS, Perina DG (2012). Out-of-hospital cardiac arrest. Emerg Med Clin North Am.

[64] Nielsen N (2012). Predictive scores, friend or foe for the cardiac patient. Resuscitation.

[65] Goto Y, Maeda T, Goto Y (2014). Decision tree model for predicting outcomes after out-of-hospital cardiac arrest in the emergency department. Crit Care.

[66] McNally B, Robb R, Mehta M, Vellano K, Valderrama AL, Yoon PW (2011). Out-of-hospital cardiac arrest surveillance -Cardiac Arrest Registry to Enhance Survival (CARES), United States, October 1, 2005-December 31, 2010. MMWR Surveill Summ.

[67] He ZJ, Ning B, Zhang ZC (2014). Build China's own emergency days-Platinum ten minutes emergency first aid activities in Japan. Chin J Crit Care Med.

[68] He ZJ, Wang LX (2013). Exploration of life path improve emergency server system mangement. Chin J Cri Care Med.

[69] He Z, Ma JX, Wang YG (2006). Establish the concept of platinum 10 minutes to improve the survival rate of sudden death in the community. Med Philos.

